# Determination of a microRNA signature of protective kidney ischemic preconditioning originating from proximal tubules

**DOI:** 10.1038/s41598-021-89195-3

**Published:** 2021-05-10

**Authors:** Usman Khalid, Robert H. Jenkins, Robert Andrews, Gilda Pino-Chavez, Benjamin C. Cossins, Rafael Chavez, Timothy Bowen, Donald J. Fraser

**Affiliations:** 1 Wales Kidney Research Unit, School of Medicine, College of Biomedical & Life Sciences, Cardiff University, Heath Park Campus, Cardiff, CF14 4XN UK; 2 Cardiff Transplant Unit, University Hospital of Wales, Cardiff, CF14 4XW UK; 3 Systems Immunity University Research Institute, Cardiff University, Cardiff, CF14 4XN UK

**Keywords:** Transcriptomics, Non-coding RNAs, miRNAs, Small RNAs, Epigenetics

## Abstract

Ischemic preconditioning (IPC) is effective in limiting subsequent ischemic acute kidney injury in experimental models. MicroRNAs are an important class of post-transcriptional regulator and show promise as biomarkers of kidney injury. We evaluated the time- and dose-dependence of benefit from IPC in a rat model of functional (bilateral) ischemia–reperfusion injury (IRI). We found optimal protection from subsequent injury following short, repetitive sequences of preconditioning insult. We subsequently used hybridization array and microRNA sequencing to characterize microRNA signatures of protective IPC and of IRI. These approaches identified a profile of microRNA changes consequent on IRI, that were limited by prior IPC. To localize these signals within the kidney, we used laser capture microdissection and RT-qPCR to measure microRNA abundance in nephron segments, pinpointing microRNA changes principally to glomeruli and proximal tubules. Our data describe a unique microRNA signature for IRI in the rat kidney. Pulsatile IPC reduces kidney damage following IRI and diminishes this microRNA signal. We have also identified candidate microRNAs that may act as biomarkers of injury and therapeutic targets in this context.

## Introduction

The kidney is a highly metabolically active organ, second only to the brain in terms of oxygen consumption. Acute Kidney Injury (AKI), to which ischemia is a predominant contributor, is a common and serious problem among hospital inpatients^1,2^. Some clinical scenarios entail a particularly high risk of ischemic AKI, these include patients undergoing cardiac surgery^3^, and cadaveric kidney transplantation in which ischemic damage to the transplanted organ prior to transplantation is a key determinant of outcome^4,5^. More recently, in the recent COVID-19 pandemic era, up to 25% of patients with SARS-Cov-2 infection develop AKI^6^. To date, no specific therapies exist to prevent AKI in those at risk^7^. Detection of the extent of ischemic kidney injury and subsequent AKI risk is challenging with currently available biomarkers^8^.

Preconditioning of an organ with brief periods of ischemia limits subsequent ischemia-induced necrosis in experimental in vivo models^9^, but it has proved challenging to translate this into a beneficial therapy^10^. This lack of translation may in part reflect the difficulty of titrating “dose” of IPC in a heterogenous patient population, and that the mechanisms underlying the protective effect have not been determined.

MicroRNAs are important post-transcriptional regulators of gene expression that help define tissue response to hypoxia and tissue injury^11–14^. Several characteristics have led to their advancement as potential biomarkers, including their ubiquitous expression, mechanistic importance in regulating key cellular processes, amenability to detection by sensitive techniques reliant on nucleic acid amplification, and resistance to degradation in tissues and fluids^15–19^. In the kidney, microRNAs are linked to multiple pathological processes^14,20,21^. MicroRNA expression may be quantified by multiple techniques developed for nucleic acid analysis, including profiling by hybridization- and microRNA-sequencing-based techniques, however existing profiling approaches may introduce significant bias^22–24^.

Here we sought to determine a definitive microRNA signature of IRI and IPC in the kidney by comparison of hybridization array and microRNA sequencing-based profiles. We then localized microRNA changes to specific components of the kidney through use of laser capture microdissection, and related changes in microRNA expression to those seen in previously determined biomarker evaluations in urine following kidney injury.

## Results

### Developing the IPC model and comparing effects of various IPC regimens

Many studies of IRI in the kidney are limited by a unilateral injury approach, which does not model AKI effectively since autoregulatory responses in the unaffected contralateral kidney maintain functions including waste excretion and sodium/water homeostasis. We therefore selected a model of bilateral IRI in the rat, in which there is functional as well as structural renal impairment following injury. Injury was elicited by clamping of both renal pedicles for 45 min of warm ischemia, following which animals were recovered and observed for two days, then culled. Forty-five minutes of bilateral IRI in the rat caused marked histological damage at 48 h when compared with sham controls, exemplified by acute tubular necrosis, endothelial disruption, glomerular changes and tubulointerstitial inflammation. A significant rise in serum creatinine (76.63 (± 13.36) μmol/L in IRI vs 31.90 (± 0.25) μmol/L in sham (p = 0.0067)) was observed, in addition to elevations in whole kidney tissue markers of kidney injury (neutrophil gelatinase-associated lipocalin, NGAL, 14-fold versus control, and kidney injury molecule-1, KIM-1, 150-fold versus control, p < 0.0001).

**Figure 1:**
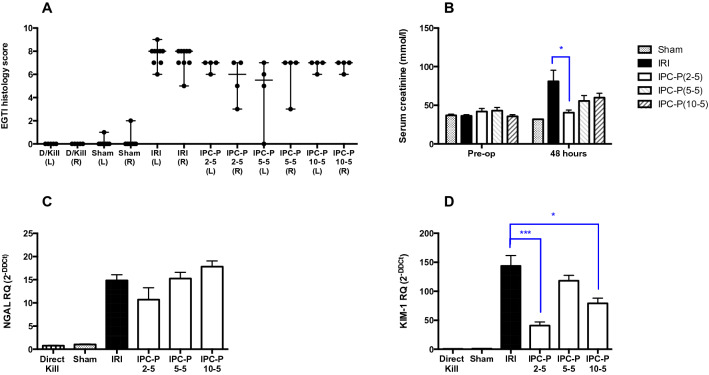
Effect of localised pulsatile IPC on EGTI score, serum creatinine, and renal injury markers NGAL and KIM-1. **(A)** Renal cortex sections were prepared from kidneys harvested 48 h after recovery from 45 min bilateral IRI, and after 3 localized pulsatile IPC regimens (comprising 3 cycles of 2, 5, or 10 min ischaemia alternated with 5 min of reperfusion (labelled IPC-P 2–5, IPC-P 5–5, and IPC-P 10–5 respectively) prior to IRI, Sections were stained with H&E and scored for Endothelial, Glomerular, Tubular, and Interstitial cell damage. EGTI Histology scores are plotted as median and range. **(B)** Serum creatinine was measured pre-operatively and at 48 h, and is plotted as mean ± SEM. **(C,D)** RT-qPCR analysis of NGAL and KIM-1 were performed following RNA extraction from whole kidney tissue. Expression is normalized to GAPDH and plotted as mean ± SEM. Numbers of animals in each group: Direct Kill (n = 5), Sham (n = 8), IRI (n = 9), IPC-P 2–5 (n = 4), IPC-P 5–5 (n = 4), and IPC-P 10–5 (n = 4). Statistical significance: *p < 0.05, **p < 0.01, ***p < 0.001.

Pulsatile approaches to preconditioning were evaluated, employing three cycles of 2, 5, and 10 min ischemia interspersed with 5 min recovery (Fig. 1). Histological evidence of kidney injury was still evident, but functional and kidney injury biomarker responses were minimized, with maximum benefit seen in animals treated with two minutes ischemia per cycle of preconditioning. Mean serum creatinine at 48 h was 40.43 (± 3.35) μmol/L, 55.65 (± 6.88) μmol/l, and 59.75 (± 5.65) μmol/L in the ‘IPC-P 2–5’, ‘IPC-P 5–5’, and ‘IPC-P 10–5’ groups compared with 76.63 (± 13.36) μmol/L in the IRI group (p = 0.014, p = 0.339 and p = 0.435 respectively). The mRNA expression of NGAL was not increased or decreased significantly compared with the IRI group (p = ns). The mRNA expression of KIM-1 was decreased twofold in the ‘IPC-P 2–5’ and ‘IPC-P 10–5’ groups compared with the IRI group (p = 0.0009 and p = 0.026 respectively), however it did not change significantly in the ‘IPC-P 5–5’ group compared with the IRI group (p = 0.359).

**Figure 2:**
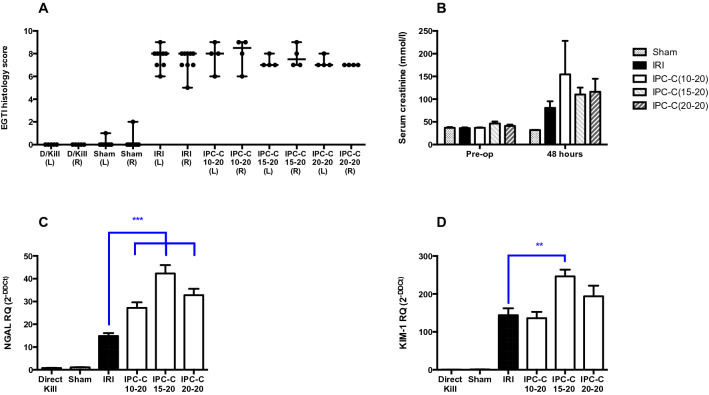
Effect of continuous IPC on EGTI score, serum creatinine and renal injury markers NGAL and KIM-1. **(A)** Renal cortex sections from Direct Kill, Sham, 45 min bilateral IRI, and 3 localized continuous IPC regimes (10, 15 and 20 min ischaemia and 20 min reperfusion (IPC-C 10–20, IPC-C 15–20, and IPC-C 20–20 respectively) prior to IRI, in rats at 48 h after reperfusion, were stained with H&E and scored for Endothelial, Glomerular, Tubular, and Interstitial cell damage. EGTI Histology scores are plotted as median and range. **(B)** Serum creatinine was measured pre-op and at 48 h and is plotted as mean ± SEM. **(C,D)** RT-qPCR analysis of NGAL and KIM-1 was performed following RNA extraction from whole kidney tissue. Expression is normalized to GAPDH and plotted as mean ± SEM. Numbers of animals in each group: Direct Kill (n = 5), Sham (n = 8), IRI (n = 9), IPC-C 10–20 (n = 4), IPC-C 15–20 (n = 4), and IPC-C 20–20 (n = 4). Statistical significance: *p < 0.05, **p < 0.01, ***p < 0.001.

Continuous preconditioning regimens using times of 10, 15 and 20 min were also investigated (Fig. 2). This led to a more pronounced subsequent AKI phenotype following IRI, with clearer histological changes, and larger rises in biomarkers of diminished kidney function (serum creatinine) and cellular injury in the kidney (KIM-1 and NGAL). Mean serum creatinine at 48 h was 154.5 (± 73.79) μmol/L, 110.2 (± 15.21) μmol/L, and 115.9 (± 28.83) μmol/L in the ‘IPC-C 10–20’, ‘IPC-C 15–20’, and ‘IPC-C 20–20’ groups compared with 76.63 (± 13.36) μmol/L in the IRI group (p = ns). The mRNA expression of NGAL was increased at least twofold in each of the continuous IPC groups compared with IRI alone (p < 0.0001). Expression of KIM-1 mRNA was increased twofold in the ‘IPC-C 15–20’ group compared with the IRI group (p = 0.0018), but not significantly increased in ‘IPC-C 10–20’ and ‘IPC-C 20–20’ groups.

**Figure 3:**
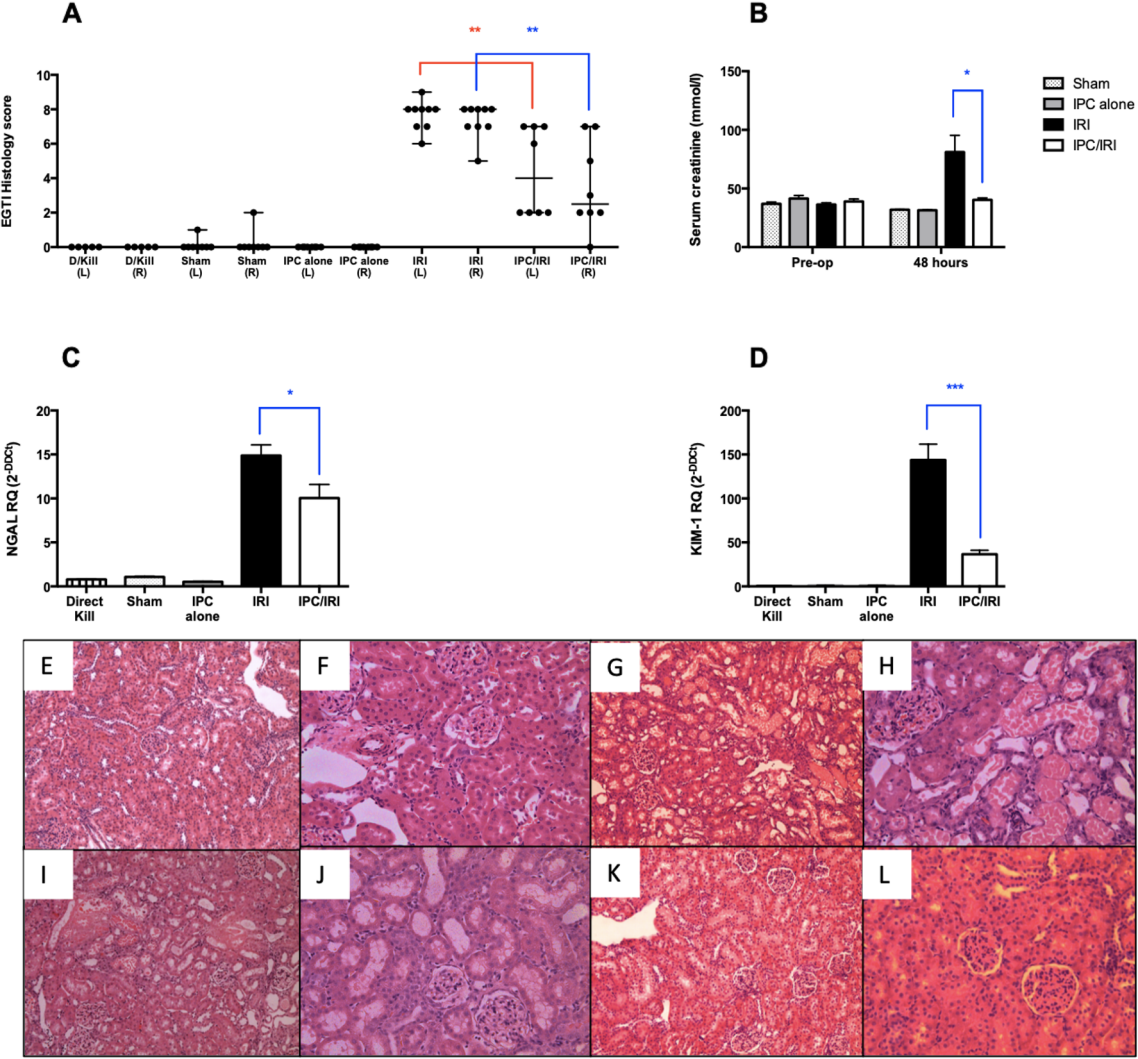
Effect of IPC on EGTI score, serum creatinine and renal injury markers NGAL and KIM-1. **(A)** Renal cortex sections from Direct Kill, Sham, 45 min bilateral IRI, localized pulsatile IPC (3 cycles of 2 min ischaemia and 5 min reperfusion) prior to IRI, and localized pulsatile IPC (3 cycles of 2 min ischaemia and 5 min reperfusion) alone without any subsequent IRI, in rats at 48 h after reperfusion were stained with H&E and scored for Endothelial, Glomerular, Tubular, and Interstitial cell damage. EGTI Histology scores are plotted as median and range. **(B)** Serum creatinine was measured pre-op and at 48 h and is plotted as mean ± SEM. **(C,D)** RT-qPCR analysis of NGAL and KIM-1 was performed following RNA extraction from whole kidney tissue. Expression is normalized to GAPDH and plotted as mean ± SEM. Renal cortex H&E micrographs (× 100 and × 200) show normal appearance in sham **(E,F)**, moderate to significant damage in IRI **(G,H)**, mild to moderate damage in IPC/IRI **(I,J)** and no damage/normal appearance in IPC alone **(K,L)** groups respectively. Numbers of animals in each group: Direct Kill (n = 5), Sham (n = 8), IRI (n = 9), IPC alone (n = 8), and IPC/IRI (n = 8). Statistical significance: *p < 0.05, **p < 0.01, ***p < 0.001.

The above data demonstrated maximal beneficial effect with the pulsatile preconditioning regimen ‘IPC-P 2–5’, which was therefore selected for microRNA profiling. This IPC regime was subsequently performed in eight independent replicates, where reduction in histological injury was seen together with reduction in biomarkers of diminished kidney function (Fig. 3) (Mean serum creatinine: sham 31.90 (± 0.25), IPC Alone 31.45 (± 0.32), IRI 76.63 (± 13.36), IPC-IRI 40.31 (± 1.59) μmol/L (p = 0.023 IRI vs IPC-IRI)) and renal injury (Mean relative expression of NGAL: Direct Kill 0.78 (± 0.07), sham 1.05 (± 0.08), IPC alone 0.51 (± 0.06), IRI 14.87 (± 1.22), IPC-IRI 10.05 (± 1.55) (p = 0.0195 IRI vs IPC-IRI); Mean relative expression of KIM-1: Direct Kill 0.54 (± 0.07), sham 1.08 (± 0.11), IPC alone 0.89 (± 0.43), IRI 143.8 (± 18.01), IPC-IRI 36.3 (± 4.40) (p < 0.0001 IRI vs IPC-IRI)). 5 of 8 animals from each group were randomly selected for microRNA profiling.

### Evaluating the microRNA expression profile of renal ischemic injury, and changes effected by ischemic preconditioning

MicroRNA sequencing and hybridisation array are widely employed methods to analyse microRNA expression. Bias is evident with both techniques when compared systematically, so we performed combined microRNA expression profiling using both microRNA sequencing and hybridisation array.

**Figure 4:**
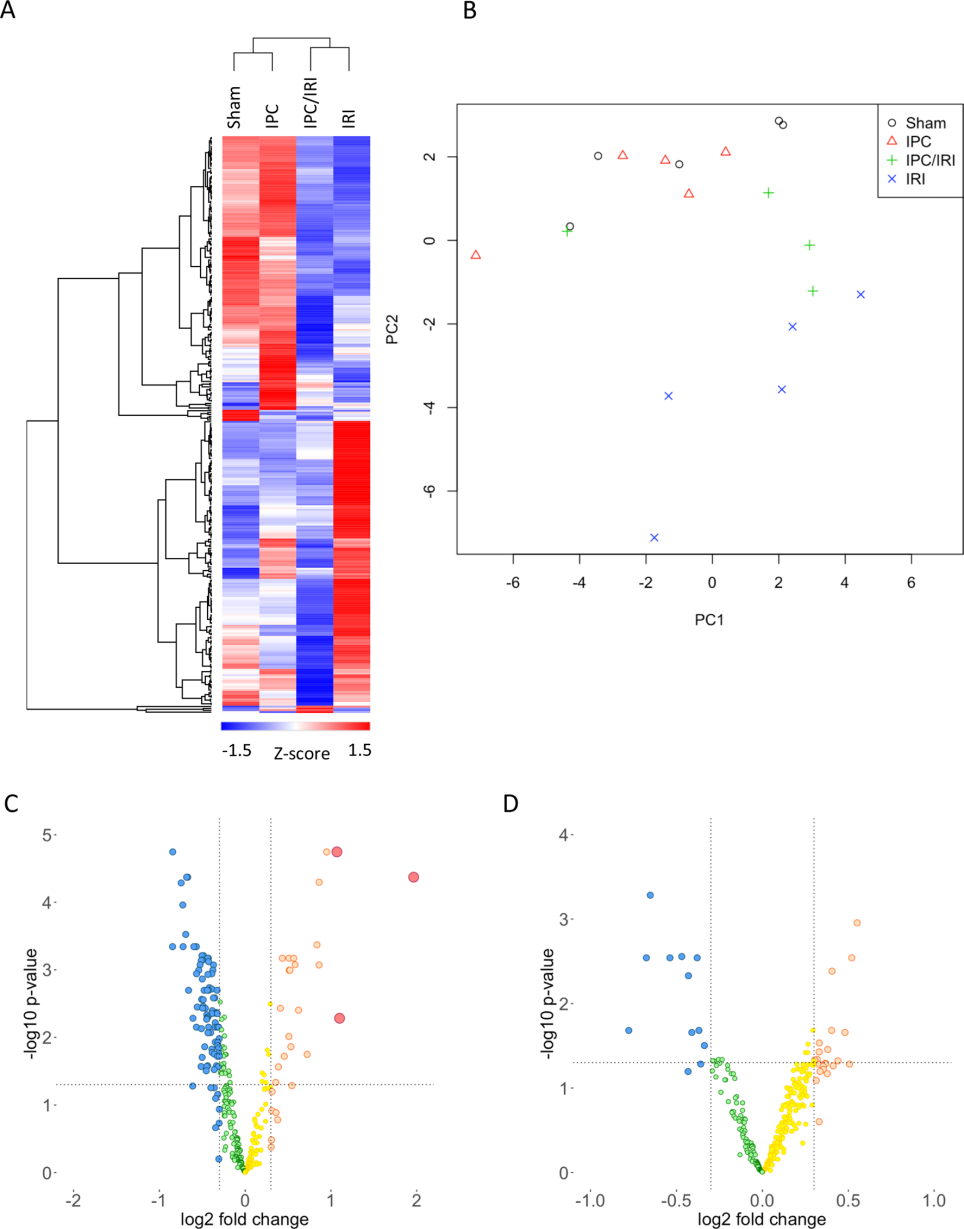
MicroRNA profiling (Microarray). Hybridisation array profiling of microRNAs from whole kidney tissue of bilateral sham, 45-min IRI, IPC alone, and IPC/IRI in rats at 48 h after reperfusion. **(A)** Principal Component Analysis. **(B)** Heat Map with unsupervised hierarchical clustering. **(C,D)** Volcano plots using thresholds for analysis of log_2_ fold-change of > 0.3 and adjusted p-value < 0.05 **(C)** ‘IRI v Sham’ and **(D)** ‘IPC/IRI v IRI’, displaying 311 detected microRNAs. Numbers of animals in each group: Sham (n = 5), IRI (n = 5), IPC alone (n = 5), and IPC/IRI (n = 5).

Hybridization array (Fig. 4) showed expression of 311 microRNAs above the prespecified detection threshold (> = 1.2 times the 25th centile of average signal intensity on >  = 2 microarray slides). Principal Component Analysis and unsupervised bicluster analysis demonstrated clear separation of expression profiles of kidneys from sham- and IRI-treated animals, and intermediate profiles in IPC-IRI animals. Prespecified thresholds for analysis were log_2_ fold-change of greater than 0.3 and significance corrected for multiple testing of p < 0.05. Comparing results from sham operated- and IRI- animals, 97 microRNAs met these thresholds, 21 upregulated and 76 downregulated. When comparing IRI vs IPC-IRI, 21 differentially expressed microRNAs were identified, 11 upregulated and 10 downregulated.

**Figure 5:**
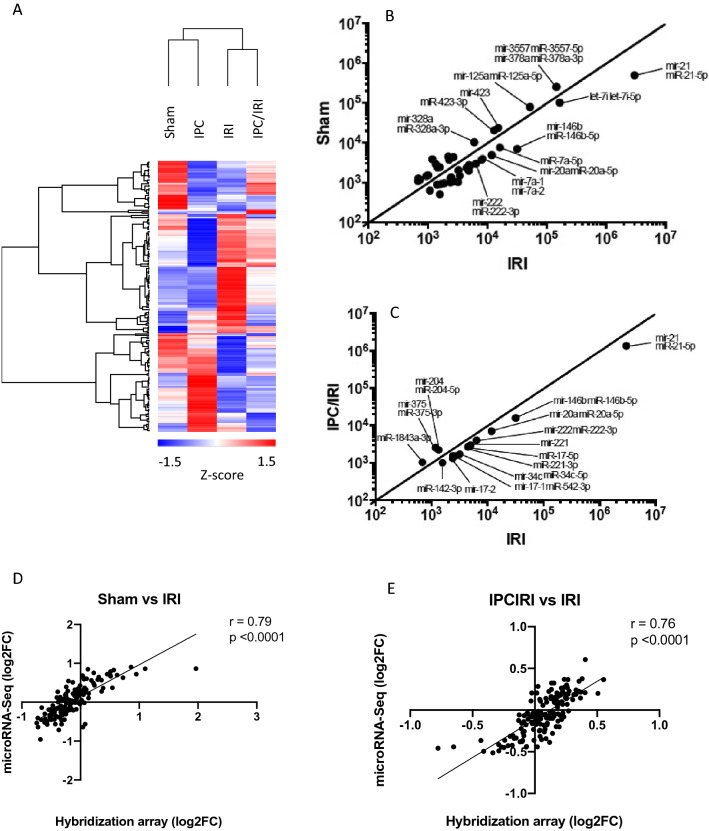
MicroRNA profiling (NGS and comparison with Microarray). Next Generation Sequencing (NGS) analysis of microRNAs from whole kidney tissue of bilateral sham, 45-min IRI, IPC alone, and IPC/IRI groups at 48 h after reperfusion. **(A)** Hierarchical clustering performed with GENE-E software **(B,C)** XY plots of **(B)** Sham versus IRI **(C)** IRI versus IPC/IRI. **(D,E)** Comparison of NGS and hybridisation array findings. Correlation plots comparing expression in NGS and Hybridization array datasets for **(D)** sham versus IRI and **(E)** IRI versus IPC-IRI groups.

MicroRNA Sequencing (Fig. 5A–C) identified 403 microRNAs as expressed (read count > 500) in kidney. Thresholds of read count > 1000 and fold change > 1.5 were applied, finding 18 upregulated and 19 downregulated microRNAs in IRI compared to sham animals. Comparing IRI- with IPC-IRI-animals, microRNA sequencing showed 9 microRNAs downregulated in IPC-IRI, and 3 upregulated.

**Figure 6:**
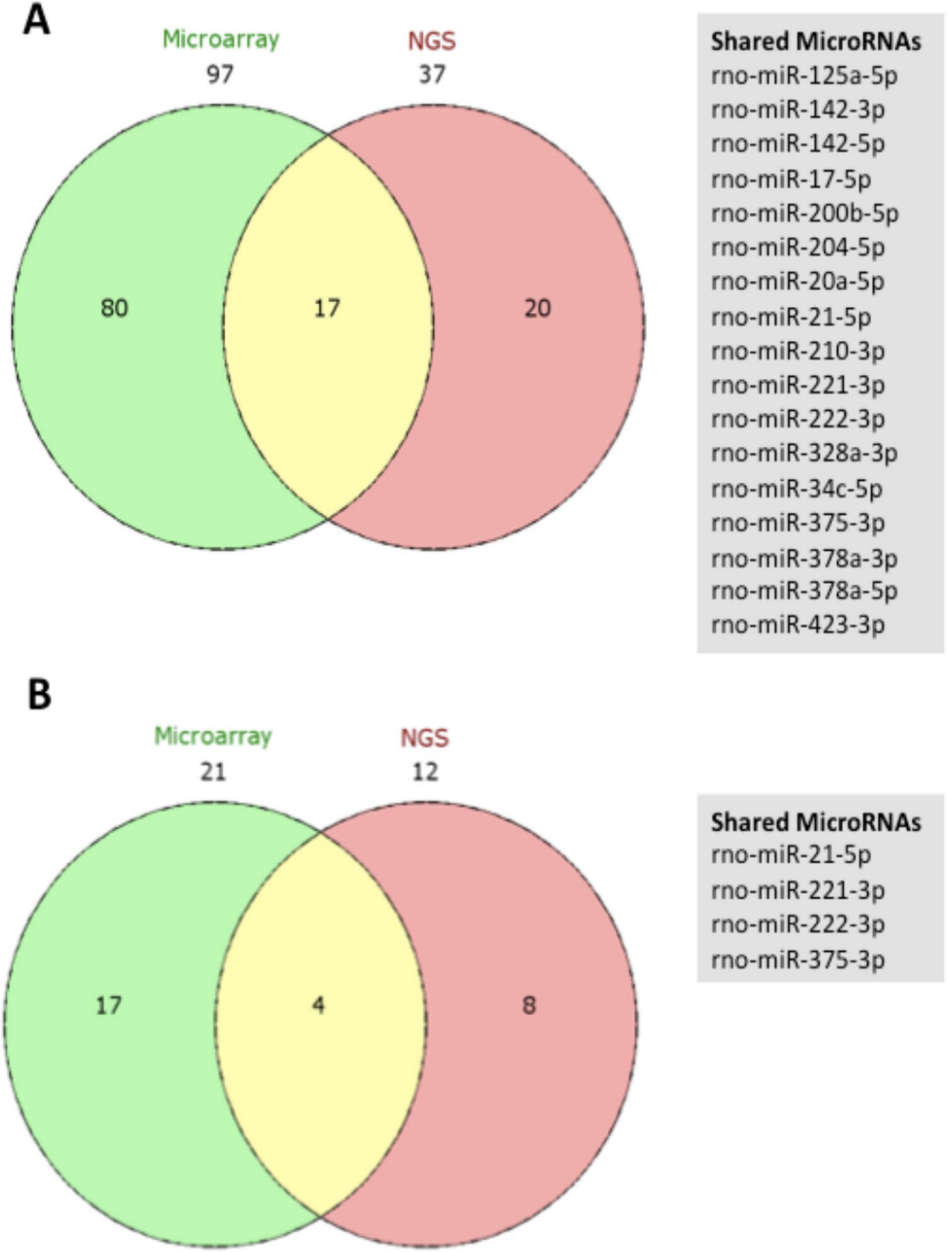
Comparison of Microarray and Next Generation Sequencing analyses. RNA extracted from whole kidney tissue of bilateral sham, 45 min IRI, IPC alone, and IPC-IRI in rats at 48 h after reperfusion underwent Exiqon Microarray (n = 5 each) and Next generation Sequencing (NGS) analyses (pooled samples n = 1 each). Venn diagrams **(A)** and **(B)** show the overlap between Microarray and NGS of differentially expressed microRNAs in ‘Sham v IRI’ and ‘IRI v IPC/IRI’ respectively.

Profiles from hybridisation arrays and microRNA sequencing were compared (Fig. 5D,E). Correlation was observed in data comparing expression in sham- and IRI-groups (r = 0.79, p < 0.0001) and between IRI and IPC-IRI groups (r = 0.76, p < 0.0001). Therefore, microRNAs were selected for further analysis based on concordant change across profiling platforms. The data demonstrated 17 microRNAs as significantly different by both techniques in IRI- versus sham samples, and for the 4 microRNAs in IPC-IRI versus IRI samples (Fig. 6). The four microRNAs different in IPC-IRI vs. IRI were also identified in sham-IRI comparison, and have concordant direction of change across both analyses (upregulated in IRI vs. Sham and IRI vs. IPC/IRI: mir-21-5p, -221-3p, -222-3p, downregulated in IRI vs. Sham and IRI vs. IPC-IRI: miR-375-3p).

### Localisation of ischemic- and protective-signal

**Figure 7:**
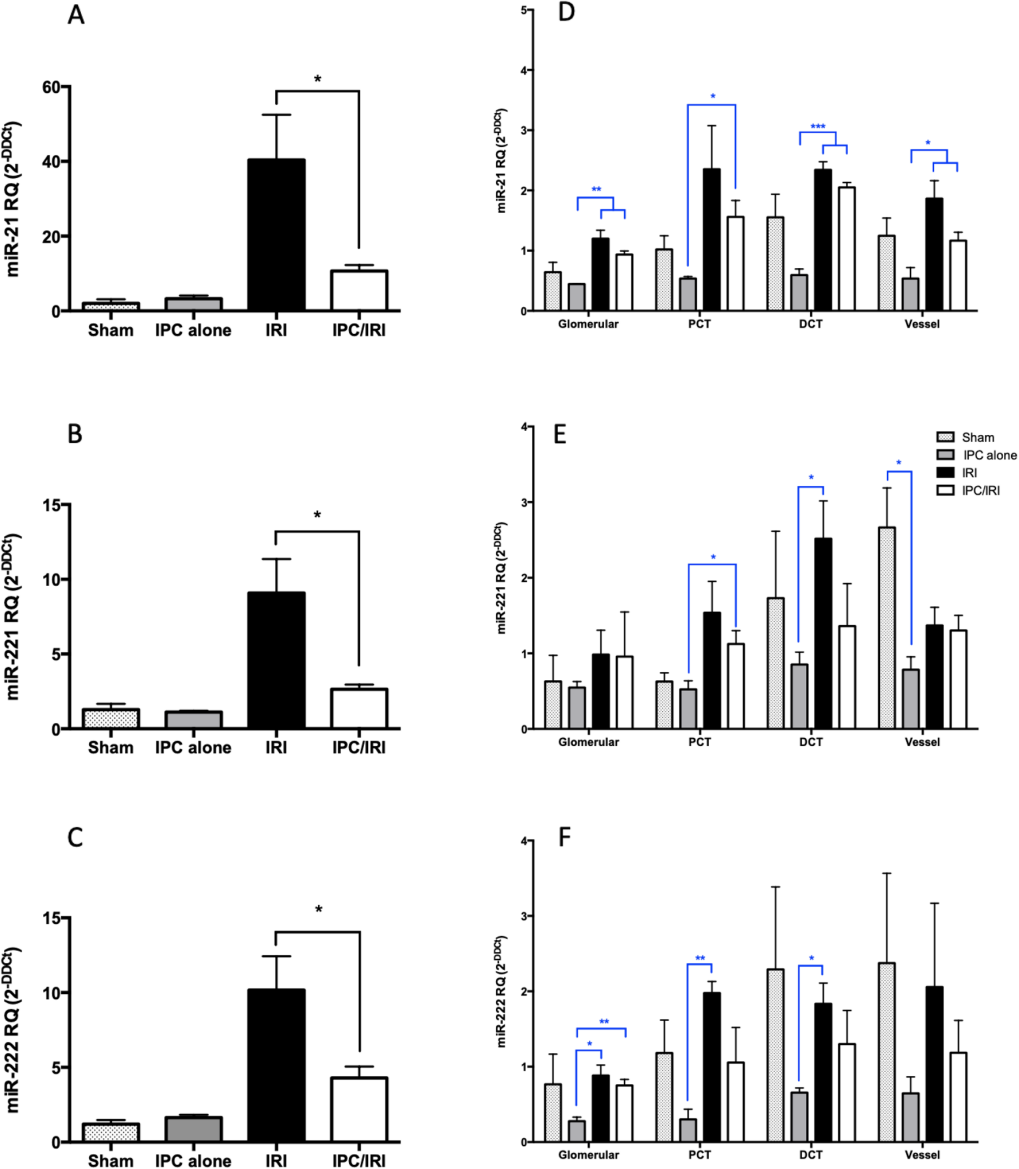
Localisation of miR-21-5p, miR-221-3p, and miR-222-3p by Laser Capture Microdissection analysis. **(A–C)** RT-qPCR for selected microRNAs in sham (n = 8), 45 min bilateral IRI (n = 9), localised pulsatile IPC prior to IRI (n = 8), and localised pulsatile IPC alone (n = 8), rats at 48 h after reperfusion **(A)** miR-21-5p, **(B)** miR-221-3p, **(C)** miR-222-3p **(D–F)** MicroRNA expression in nephron sub-compartments. Laser Capture Microdissection (LCM) of: Glomerulus, G; Proximal Convoluted Tubule, PCT; Distal Convoluted Tubule, DCT; and Vessel, V using cortical sections from Sham, IRI, IPC prior to IRI, and IPC alone (n = 3 animals per group). **(D)** miR-21-5p, **(E)** miR-221-3p, **(F)** miR-222-3p. Expression is normalised to miR-16-5p and plotted as mean ± SEM. Statistical significance: *p < 0.05, **p < 0.01, ***p < 0.001. Statistically significant differences between groups have been indicated.

The above data identify a profile of microRNAs differentially expressed in kidney following IRI and diminished in kidneys protected by IPC prior to IRI. Four microRNAs that were significantly differentially expressed by IRI and IPC (3 upregulated by IRI and diminished by IPC (miR-21-5p, -221-3p, and -222-3p), 1 down regulated by IRI and upregulated in IPC (miR-375-3p)) in both hybridisation array and microRNA Sequencing expression profiles were chosen for further analysis. Upon confirmatory RT-qPCR, miR-375-3p expression was extremely low with very high CT values (CT > 35), and therefore was excluded from further analyses. Profiling data from whole kidney is derived from a complex admixture of cells. To identify predominant locations of microRNA changes, we performed Laser Capture Microdissection (LCM) to obtain RNA derived from four major anatomical components of the kidney: glomeruli, proximal tubules, distal tubules, and vessels, and evaluated individual microRNAs by RT-qPCR. Basal expression of microRNAs-21-5p, -221-3p and –222-3p was observed in all four components. Ubiquitously high basal miR-21-5p expression was further upregulated in each following IRI, while miR-221-3p and –222-3p were basally abundant in vessels and were upregulated particularly in tubules following IRI (Fig. 7).

## Discussion

Here, we have systematically examined the ability of ischemic preconditioning to limit subsequent AKI due to ischemic injury. The data demonstrated optimal protective effect with a pulsatile approach, using three cycles of short duration ischemia and reperfusion. Optimal protective effect was dependent on both duration and timing of preconditioning insult. Subsequently, we profiled microRNAs in the kidney by both hybridisation array and microRNA sequencing. These approaches uncovered a microRNA signature of kidney IRI in the rat that was diminished by IPC, suggesting that it is an injury signature. Finally, using laser capture microdissection and quantitative RT-qPCR, we examined localisation of selected microRNAs identified in profiling.

Ischemic preconditioning has been widely reported as beneficial in limiting subsequent ischemic damage to the kidney in experimental models of renal injury^9^. However, benefit is not universal, with some studies reporting worsening of subsequent injury, and a failure to translate into benefit in clinical trials thus far^10^. Here, we have systematically evaluated ischemic preconditioning approaches in a rat model of ischemic injury. IRI leading to AKI is typically bilateral and important differences are observable in renal response to ipsilateral vs. contralateral renal ischemia. Therefore, a bilateral model of IRI was employed. We found that IPC was effective in protecting from subsequent IRI, but that this protective effect was dependent on duration and timing of preconditioning insult, such that a short duration of preconditioning applied repeatedly with recovery intervals was optimal.

We then determined the microRNA expression profile of IRI, and of effective bilateral preconditioning. We performed microRNA profiling using two distinct approaches, hybridization array and microRNA sequencing, and further evaluated abundance of selected microRNAs by RT-qPCR. Our data show overall concordance in detected changes in expression pattern in response to IRI. There were important differences in apparent relative abundance of microRNAs in the profiles obtained from the different techniques, consistent with the suggestion that these different approaches exhibit distinct biases in ease of detection of short RNA sequences^22–24^.

The microRNA profile seen in the kidney following IRI was blunted in kidneys protected by IPC, suggesting that the IRI pattern is an injury signature^25^. Laser capture microdissection demonstrated a strong tubular upregulation in microRNAs-21-5p, -221-3p and -222-3p in response to injury. Renal tubules are the single most abundant contributor to overall kidney mass. Highly metabolically active, they are thus susceptible to ischemic damage. We have recently described microRNA profiling of urine from patients following cadaveric kidney transplantation, in which circumstances the transplanted kidney may suffer ischemic injury leading to delayed function^19^. Notably, the four microRNAs identified by both hybridisation array and microRNA sequencing in the current paper as significantly upregulated by IRI , and blunted in response by IPC, are increased in expression in the urine of kidney transplant recipients exhibiting delayed recovery of kidney function due to ischemic injury^19^. We conclude that detection of these microRNAs in urine may serve as biomarkers of ischemic injury to kidney tubules.

miR-21-5p is abundantly expressed in the kidney and has been linked to renal pathology by prior studies. Upregulation of this transcript has been linked to apoptosis^26,27^, autophagy^28^, IPC^29,30^ and has been proposed as a biomarker of kidney injury^31^. Here, miR-21-5p was significantly increased in IRI, and reduced by IPC, supporting its role as a reliable and useful biomarker of IRI-mediated kidney injury. In other studies, however, miR-21-5p expression was increased by IPC. Xu et al. showed that 15 min IPC protected the kidney from injury 4 days later, termed delayed IPC^29^. This delayed IPC upregulated miR-21-5p expression, and was thought to be protective due to the anti-apoptotic properties of miR-21-5p^29^. Another study also showed miR-21-5p upregulation was associated with the protective effects of Xenon preconditioning to the kidney^30^. A recent study observed that delayed IPC associated with upregulation of miR-21-5p protected the kidney from injury via HIF-1α by inhibiting its target prolyl hydroxylase domain protein 2 (PHD2)^32^. Accumulating evidence points to significant roles for miR-21-5p in regulating both protective and pathological molecular pathways^33^.

The current data also demonstrate upregulation of miR-221-3p and miR–222-3p following ischemic injury. In contrast to miR-21-5p, these microRNAs have not previously been linked to ischemia in the kidney. Interestingly, miR-221-3p and miR-222-3p are co-located on the X Chromosome, and have the same seed region, predictive of shared regulatory targets. These microRNAs are important regulators of angiogenesis and vascular remodelling, and work in Zebrafish shows that miR-221-3p is required for vascular development^34^. They are also pro-oncogenic and linked to invasiveness and cancer cell survival, in which situation they are co-ordinately expressed. Notably, upregulation of miR-221-3p and miR-222-3p is a poor prognostic marker in renal carcinoma^35^.

For the histology and LCM, renal cortex was used, and microRNAs were detected in glomeruli, proximal tubules and vessels. The renal cortex is of particular interest in AKI to CKD transition because histological change in the cortex (tubulo-interstitial fibrosis) is a strong predictor of progression in CKD^36^. However, medullary injury is also highly evident following IRI^37^, and medullary microRNA responses may be an important area for future study. Further localisation within kidney of microRNA changes is an important consideration. Laser Capture Microdissection (LCM) is a valuable way of studying subsegments of the nephron, however an important limitation is that the samples extracted using it yield relatively small amounts of RNA, requiring a carrier RNA to facilitate RNA recovery, which may have increased variability and reduced fold-changes seen between groups, when compared to analysis of whole tissue in this study.

In summary, these data demonstrate the timing- and dose-dependence of the protective effect of IPC in a bilateral rat IRI model. They further identify a profile of microRNA changes seen in the kidney following IRI and limited by protective IPC, most notably miR-21-5p, -221-3p and –222-3p, localised particularly to renal tubuli.

## Materials and methods

### Animal experiments

All animal experiments were conducted according to the United Kingdom Use of Animals (Scientific Procedures) Act 1986, under licence PPL30/3097. Fifty-eight (58) adult (8 to 12-week-old) male Lewis rats weighing 180–220 g were used (Harlan Laboratories, UK). The rats were given a 7-day period of acclimatisation to their new surroundings, and were housed and handled according to the local institutional policies and procedures licenced by the Home Office. Ethical approval for all the protocols within the Licence was provided by the Animal Welfare and Ethical Review Body under the Establishment Licence held by Cardiff University. The ARRIVE guidelines were followed for all experiments involving use of animals. Initial experiments were performed at n = 4, to evaluate likely optimal preconditioning approach (reflecting options of preconditioning time and interval, and of pulsatile versus continuous preconditioning delivery). Based on this work, an approach of pulsatile preconditioning was selected, and power calculation from the initial dataset indicated that n = 8 per group gave acceptable power (α 0.05, β 0.8).

Analgesia was provided 24 h pre-op throughout until the time of retrieval in the form of 200 mcg of buprenorphine dissolved in 500 ml of drinking water. Each animal was given general anaesthesia using isoflurane. Under aseptic conditions, a midline laparotomy and atraumatic clamping of both renal pedicles for 45 min was performed, followed by recovery and reperfusion for 48 h before terminal anaesthesia (IRI group; n = 9). Sham controls underwent the same operation without the renal pedicle clamping (n = 8). IPC/IRI animals underwent a number of regimes: Renal pedicle clamping of 10, 15 and 20 min followed by 20 min reperfusion prior to the IRI respectively (IPC-C 10–20, IPC-C 15–20, and IPC-C-20–20, n = 4 each); and 3 cycles of 2, 5 and 10 min ischaemia and 5 min reperfusion respectively (IPC-P 2–5, IPC-P 5–5, and IPC-10–5, n = 4 each), prior to IRI. The IPC regime of 3 cycles of 2 min ischaemia and 5 min reperfusion was found to be most protective against injury, so a further 4 animals underwent surgery to complete n of 8 (IPC/IRI, n = 8). In addition to this, an extra group of animals underwent 3 cycles of 2 min ischaemia and 5 min reperfusion without any subsequent IRI (IPC Alone, n = 8). Direct Kill animals involved terminal anaesthesia, midline laparotomy, exsanguination, and retrieval of kidneys (n = 5).

### Histology and laser capture micro-dissection (LCM)

Kidney tissue was embedded in paraffin and sectioned for Haematoxylin and eosin (H&E) staining. Scoring was carried out in a blinded fashion using the Endothelial, Glomerular, Tubular, and Interstitial (EGTI) scoring system, developed specifically for animal research on kidney tissue in the context of injury^38^.

For LCM, 12 paraffin embedded kidney blocks (Sham, IRI, IPC/IRI, and IPC alone groups each with n = 3) were used to isolate glomeruli, proximal convoluted tubules, distal convoluted tubules, and vessel tissue (including endothelial cells), using the Arcturus Pixcell IIe infrared laser enabled LCM system (Applied Biosystems). These nephron subsegments were identified by an experienced Consultant Renal Histopathologist based on their recognisable morphological characteristics. Specifically, differentiation between PCT and DCT was based on tubular size, cytoplasmic density, nuclear position, type of cubical epithelial cells, and presence or absence of the brush border^39^. For each kidney block, two 6-μm sections were cut, placed in the middle third of an uncharged, uncoated glass slide (VFM White coat slides CellPath Ltd) and stained according to the method described by Espina et al.^40^. Using infrared laser, the target tissue was bonded to a polymer membrane located on a cap (Arcturus Capsure Macro LCM caps—Applied Biosystems) placed onto the slide, which when lifted removed the selected tissue.

### Renal function

Serum creatinine was measured from blood samples taken pre-op (at 0 h) and at time of retrieval (at 48 h) using the Jaffe reaction.

### RNA extraction and RT-qPCR

Whole kidney tissue was stored in RNA later solution immediately at time of retrieval and frozen at − 80 °C. Total RNA was extracted using TRIzol reagent (Life Technologies, Cat. No. 15596018) according to manufacturer’s instructions. RNA quality was assessed using the Agilent 2100 Bioanalyzer with RNA 6000 Nano chips (Santa Clara, CA, USA) and quantified prior to RT-qPCR. cDNA was generated using a High Capacity Reverse Transcription Kit (Life Technologies, Cat. No. 4368814) with random primers or specific stem loop primer for the TaqMan miRNA assays. RT-qPCR was performed on a ViiA7 Fast Real-Time PCR System (Life Technologies). NGAL and KIM-1 were quantified by POWER SYBR GREEN PCR Master Mix (Life Technologies, Cat. No. 4368706) with gene-specific primers, normalised to GAPDH, as previously described^41^.

RNA extraction from LCM samples was performed from the polymer membrane on the LCM caps, using the RecoverALL Total Nucleic Acid Kit (Ambion, Cat. No. AM1975) according to the manufacturer’s recommendations and protocol. Two modifications were made to the protocol: (1) The deparaffinisation step has been performed before the LCM procedure and therefore was omitted; and (2) 1 μg of RNA carrier (MS2 RNA, Roche, Sigma-Aldrich Ltd, Cat. No. 10165948001) was added at the nucleic acid isolation stage.

Selected microRNAs (miR-21-5p, miR-221-3p, miR-222-3p) were quantified by commercially available TaqMan miRNA assays (suitable for rat microRNAs) according to the manufacturer’s instructions and their expression normalized to miR-16-5p. TaqMan miRNA gene expression assay ID numbers were hsa-miR-16-5p (000391), hsa-miR-21-5p (000397), hsa-miR-221-3p (000524), and hsa-miR-222-3p (002276) (Life Technologies, Cat. No. 4427975). The relative changes in gene expression were analysed by the 2-DDC_T_ method^42^.

### MicroRNA profiling

#### Microarray

RNA samples extracted from the kidney tissue of 4 groups (Sham, IRI, IPC/IRI and IPC Alone, n = 5 each) were labelled using the miRCURY LNA microRNA Hi-Power Labelling Kit, Hy3/Hy5 and hybridised on the miRCURY LNA microRNA Array (7th Gen) following a dual-colour experimental design. The threshold of detection was calculated for each individual microarray slide as 1.2 times the 25th percentile of the overall signal intensity of the slide. MicroRNAs with intensities above threshold in less than 20% (or 2) of the samples were removed from the final dataset used for expression analysis. The number of microRNAs detectable above background threshold was identified for each sample (out of a total of 714 microRNAs) and comparison between groups was made. The full dataset is accessible in the ArrayExpress database at EMBL-EBI (www.ebi.ac.uk/arrayexpress) accession number E-MTAB-9900.

#### Next generation microRNA sequencing

Next Generation Sequencing (NGS) was performed on RNA extracted from kidney tissue from 4 groups (Sham, IRI, IPC/IRI and IPC alone, pooled n = 1 for each group). The raw sequence reads were mapped to the rat assembly (Rnor_5.0) using bwa^43^ and reads assigned to miRs in miRbase^44^ (rho.gff) using featureCounts^45^. Reads were normalized in the DESeq2 package in R^46^ and pairwise comparisons performed by chi-square. Hierarchical clustering on normalized data was performed using the GENE-E software and marker selection used to interrogate the pair-wise comparisons. To interpret the data set, the normalised read counts were filtered to exclude values below 500, and the data set was filtered to exclude fold change values of < 1.5 increase and decrease. The new data set was then tabulated and visualised on XY plots. The full dataset is accessible in the ArrayExpress database t EMBL-EBI (www.ebi.ac.uk/arrayexpress) accession number E-MTAB-9896.

### Statistical analysis

Statistical analyses were performed using GraphPad Prism version 6 for Macintosh software (GraphPad Software, San Diego, CA). Data were expressed as either median (and range) or mean (± SEM) and assessed for statistical significance by one-way analysis of variance (ANOVA) if normally distributed, or Kruskal–Wallis test if not. Significance level was determined using p-value < 0.05.
